# Detection of nanoparticles suspended in a light scattering medium

**DOI:** 10.1038/s41598-021-99768-x

**Published:** 2021-10-12

**Authors:** Yan Ye, David Y. H. Pui

**Affiliations:** 1 Y2Y Technology, Santa Clara, CA 95052 USA; 2 Particle Technology Lab, Mechanical Engineering Department, Minneapolis, MN 55455 USA

**Keywords:** Nanoscience and technology, Optics and photonics, Physics

## Abstract

Intentionally intensifying the light scattering of medium molecules can allow the detection of suspended nanoparticles under conditions not suitable for conventional optical microscopies or laser particle counters. Here, we demonstrate how the collective light scattering of medium molecules and nanoparticles is imaged in response to the power, frequency, and oscillating direction of the incident light wave electric field, and how this response can be used to distinguish between nanoparticles and microparticles, such as viruses or bacteria. Under conditions that the medium light scattering is intensified, suspended nanoparticles appear as magnified shiny moving dots superimposed on the quasi-steady background of medium light scattering. Utilizing the visual enlargement resulted from the enhanced light scattering and possible light interference, we can detect directly suspended nanoparticles that are much smaller than visible light wavelengths even in unopened water bottles or other large containers. This suggests new approaches for detecting nanoparticles with many potential applications.

## Introduction

**Figure 1:**
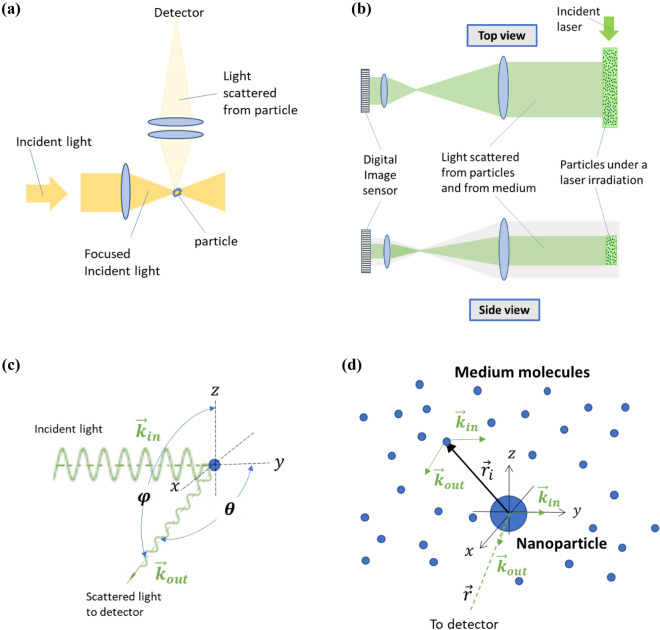
Optical diagrams and vectors of incident light and scattered light. (**a**) An optical diagram for the method derived from the development of ultramicroscopes in the early twentieth century^4^, which is a dark field approach with condenser and objective lenses to observe or detect a particle in the optical focus. This approach continues to be the basic mechanism of many advanced optical microscopes and laser particle counters used today. (**b**) An optical diagram for the proposed method to detect suspended nanoparticles contained in a large container utilizing medium light scattering. Image of light scattering by the nanoparticles and by the medium under the irradiation of a collimated laser beam is detected by an image sensor with an afocal telephoto setup. Both the top view and the side view is shown to present beam coverage. (**c**) Wave vectors for detecting the light scattered by a particle or molecule at an angle $\mathrm{\varphi }$ to the direction of the oscillating dipole moment or at an angle $\uptheta$ to the direction of the laser propagation. (**d**) Wave vectors and position vectors for a light scattering unit in which a suspended nanoparticle is surrounded by *n* light scatting medium molecules. The nanoparticle is located at the origin of the coordinate system, $\overrightarrow{{r}_{i}}$ is the vector between the nanoparticle and a medium molecule, and $\overrightarrow{r}$ is the vector between the nanoparticle and the detector.

Light scattering has been widely used for detecting suspended particles in gaseous and liquid media^1,2^. To detect particles smaller than the wavelength of the light source, the technique is limited to a small optical focus^3,4^, as shown in Fig. 1a. The detection of suspended nanoparticles remains a challenge, especially those in containers where the optical focusing settings are not suitable. The development of nanoparticle detection has been desperately needed for various purposes, such as checking the cleanliness of unopened bottled drinking water, checking the particulate contaminants directly in semiconductor fabrication or other sensitive equipment, and even checking the stability of vaccines in sealed bottles for verification of their expiration date. To break through the limitations of existing instruments and be able to directly detect suspended nanoparticles contained in large containers, it is necessary to develop an alternative mechanism to magnify the particles, rather than just relying on the optical lenses. This work proposes a method to detect suspended nanoparticles under the condition that the light scattering by medium molecules is sufficiently intensified. Under this condition, light scattering by suspended particles is more enhanced and may constructively interfere with the light scattered by the medium. Therefore, a particle image detection approach, as shown in Fig. 1b, becomes feasible to detect nanoparticles that are much smaller than the wavelength of incident light. It is possible to make an ancient visual recognition approach regain new applications with the help of new technologies.

The observation of light scattering by a medium without any suspended particles can be traced back to the mid-nineteenth century. However, the phenomenon can only be observed under certain conditions of the medium, such as critical opalescence due to strong fluctuation in the density or refractive index of the medium near the critical state^5,6^. When a medium is far away from its critical state, density fluctuations in the medium are so small that even if a focused solar light beam is used, the light scattering by the medium still cannot be noticed. Historically, light scattering is observed when fine particles are added into the pure medium or in a colloidal solution, as described by the Tyndall effect^7^. With the advent of laser technology, however, it has become easier to observe light scattering by a pure medium. In the 1970s. An Ar-ion laser was used to study light scattering by pure water to characterize its physical properties such as the light scattering depolarization and anisotropy^8^. In the last few years, lasers have become more powerful, more compact, and more cost-effective. The new developments in laser technology and image sensing technology make it possible to further utilize the light scattering of pure medium for particle detection and analysis.

When light shines on nanoparticles suspended in a medium, the charges in the nanoparticles and the medium molecules move in sync with the incoming electric field of incident light wave, resulting in a collection of induced oscillating dipoles with the same frequency and orientation as the incident electric field. Each induced oscillating dipole emits electromagnetic radiation in all directions. The intensity of the emitted radiation depends on the direction of the light scattering as described as Rayleigh scattering. From the standpoint of an individual nanoparticle or medium molecule, the wave vectors of the incident light and the scattered light to a detector are depicted in Fig. 1c. Typically, the distance between nanoparticles suspended in a medium is much larger than the incident light wavelength. It is reasonable to simulate the whole light scattering as it comes from many small scattering units, and each scattering unit has one nanoparticle of polarizability ${\alpha }_{p}$ surrounded with *n* medium molecules of polarizability ${\alpha }_{m}$, as sketched in Fig. 1d. The value *n* can be calculated using the Avogadro number and the molar mass in the corresponding volume of the medium. At the detector position, the electric fields emitted from the nanoparticle and from each medium molecule due to the far-field radiation can be expressed as ${E}_{s,p}={E}_{0}{A}_{p}{e}^{\mathrm{i}\left(\omega t-{\varphi }_{p}\right)}$ and ${E}_{s,m,i}={E}_{0}{A}_{S,i}{e}^{\mathrm{i}\left(\omega t-{\varphi }_{m,i}\right)}$ where ${\varphi }_{p}$ and ${\varphi }_{m,i}$ are the phases of the nanoparticle and the medium molecule due to wave traveling paths, ${A}_{p}=\frac{\pi {\alpha }_{p} }{r{\lambda }^{2}}sin\varphi$, ${A}_{m,i}=\frac{\pi {\alpha }_{m}}{r{\lambda }^{2}} sin\varphi$, $\varphi$ is the emitting angle of the scattered wave relative to the induced dipole moment as illustrated in Fig. 1c, and $r$ is the distance between the particle or molecule and the detector. The intensity of light scattering by each nanoparticle can be expressed as

1 $${I}_{p}=\frac{1}{2}{\varepsilon }_{0}c{{E}_{0}}^{2}{{A}_{p}}^{2}={I}_{0}\frac{{\pi }^{2}{ {\alpha }_{p}}^{2}}{{r}^{2}{\lambda }^{4}}{sin}^{2}\varphi$$

where ${I}_{0}=\frac{1}{2}{\epsilon }_{0}c{E}_{0}^{2}$ is the intensity of incident light, ${\epsilon }_{0}$ is the vacuum permittivity, and $c$ is the speed of light in the vacuum. By replacing the polarizability ${\alpha }_{p}$ with ${\alpha }_{m}$, the equation also applies to each medium molecule. Certainly, the polarizability of a medium could be much smaller than that of a suspended particle. For example, the polarizability of water^9^ is 1.47 × 10^–12^ µm^3^ and the polarizability of air^10^ is 2.12 × 10^–11^ µm^3^ which are several orders of magnitude smaller than the 4.28 × 10^–8^ µm^3^ polarizability of a 5 nm diameter dielectric particle calculated with an equation for an isotropic spheric particle^11^. Without the presence of the nanoparticle, the total intensity of light scattered by the *n* light scattering medium molecules can be expressed as ${I}_{nM}=\frac{1}{2}{\varepsilon }_{0}c{{E}_{0}}^{2}\sum_{i=1}^{n}\sum_{j=1}^{n}{A}_{m,i}{A}_{m,j}\mathrm{cos}\left({\varphi }_{S,i}-{\varphi }_{S,j}\right)$. Considering ${A}_{m,i}{\approx A}_{m,j}$ for the molecules in the small light scattering unit, the intensity becomes

2 $${I}_{nM}\approx {I}_{0}\frac{{\pi }^{2}{ {\alpha }_{m}}^{2}}{{r}^{2}{\lambda }^{4}}{sin}^{2}\varphi \sum_{i=1}^{n}\sum_{j=1}^{n}\mathrm{cos}\left({\varphi }_{S,i}-{\varphi }_{S,j}\right)$$

which has the main light scattering characteristics as the Eq. (1). To include the effect of concentration, a structure factor^12^ can be used here for better accuracy. When both the nanoparticle and *n* medium molecules are present, the total electric field reaching the detector is ${E}_{p,nM} = {E}_{s,p}+ \sum_{i=1}^{n}{E}_{s,m,i}={E}_{0}\left({A}_{p}{e}^{\mathrm{i}\left(\omega t-{\varphi }_{p}\right)}+\sum_{i=1}^{n}{A}_{m,i}{e}^{\mathrm{i}\left(\omega t-{\varphi }_{m,i}\right)}\right)$ and the intensity reaching the detector can be expressed as (see "Methods")

3 $${I}_{ p,nM}={I}_{p}+{I}_{nM}+2{I}_{p}\sum_{i=1}^{n}\frac{{A}_{S,i}}{{A}_{p}}\mathrm{cos}\left({\varphi }_{m,i}-{\varphi }_{p}\right)$$

Equation (3) shows the superposition of the light scattered by a nanoparticle with light scattered by the medium as well as the mutual interference between the scattered lights.

The light scattering intensity is a time average that covers many electric field oscillating cycles. If considering only the light scattering itself, a nanosecond period is sufficiently long because it covers approximately 500,000 cycles for light of 530 nm wavelength. However, when calculating the light scattering intensity sensed by an image sensor, the period for the average could be much longer. The frame rate of image sensors currently ranges from 10 to 1000 frames per second roughly. If an incident light source is not coherent, ${\varphi }_{M,i}$ and ${\varphi }_{p}$ can be any random number even in a short sensing time and it is likely to have the time average $\langle \mathrm{cos}\left({\varphi }_{m,i}-{\varphi }_{p}\right)\rangle =0$. When using a laser, the incident light irradiating on all small light scatters and the nanoparticle is coherent. However, due to the random motion of the medium molecules and the nanoparticle, ${\varphi }_{m,i}$ and ${\varphi }_{p}$ vary with the change of the distance between them during the sensing period. Since the number concentration of medium molecules is so high, it can be considered that the position evacuated by a molecule can be refilled by another molecule at a certain rate. The phase difference ${\varphi }_{m,i}-{\varphi }_{p}$ can be expressed by the distance ${\overrightarrow{r}}_{i}$. For $\theta =\frac{\pi }{2}$, one can therefore have ${\varphi }_{m,i}-{\varphi }_{p}={\overrightarrow{r}}_{i}\cdot \overrightarrow{q}=2\sqrt{2}\pi \frac{{r}_{i}}{\lambda }$, where $\overrightarrow{q}=$ ${\overrightarrow{k}}_{out}-{\overrightarrow{k}}_{in}=\frac{4\pi }{\lambda }\mathrm{sin}\frac{\theta }{2}$ is the scattering vector^12^. The scattering angle $\theta$ is illustrated in Fig. 1c. For the light scattering unit, the average light scattering sensed by the image sensor during a sensing period can be expressed as

4 $$\langle {I}_{ p,nM}\rangle ={I}_{p}+{I}_{nM}+2{I}_{L}\sum_{i=1}^{n}\frac{{A}_{S,i}}{{A}_{L}}{P}_{m}({r}_{i})\mathrm{cos}\left(2\sqrt{2}\pi \frac{{r}_{i}}{\lambda }\right)$$

where the probability ${P}_{m}({r}_{i})$ represents the chance of lights scattered by the medium molecules at location ${r}_{i}$ to be coherent with the light scattered by the nanoparticle during the sensing period. If the probability ${P}_{m}({r}_{i})$ is constant everywhere, there will be the first constructive interference effect in an area near the nanoparticle and the first destructive interference effect in an area further away from the nanoparticle. At the location of ${r}_{i}=\frac{\sqrt{2}}{8}\lambda$, the sub-term of the third term in Eq. (3) changes from positive to negative.

Total light scattering intensity in a scattering volume is often used to calculate the brightness of a light scattering image. When the distance between the particles suspended in a medium is larger than the wavelength of the incident laser, that is, when the particles are not highly concentrated, i.e., less than 10^12^ particles per cubic centimeter, interference between lights scattered from particles can be ignored. Under the condition, the total light scattering intensity can be calculated, in a simplified approach, by summing the intensities from all light scattering units in the scattering volume of interest. For mono-dispersed particles, the total light intensity in a light scattering volume can be expressed as

5 $${I}_{total}={n}_{p}\langle {I}_{ p,nM}\rangle$$

where ${n}_{p}$ is the number of particles in the volume. For poly-dispersed particles of *k* different particle sizes, the total light intensity in a light scattering volume can be expressed as

6 $${I}_{total}=\sum_{p=1}^{k}{n}_{p}\langle {I}_{ p,nM}\rangle$$

For poly-dispersed particles, the total light scattering intensity perceived by an image sensor is a function of the size distribution of the suspended particles. The intensity can be used further to determine the Rayleigh ratio.

## Results

### Light scattering image of a pure medium

**Figure 2:**
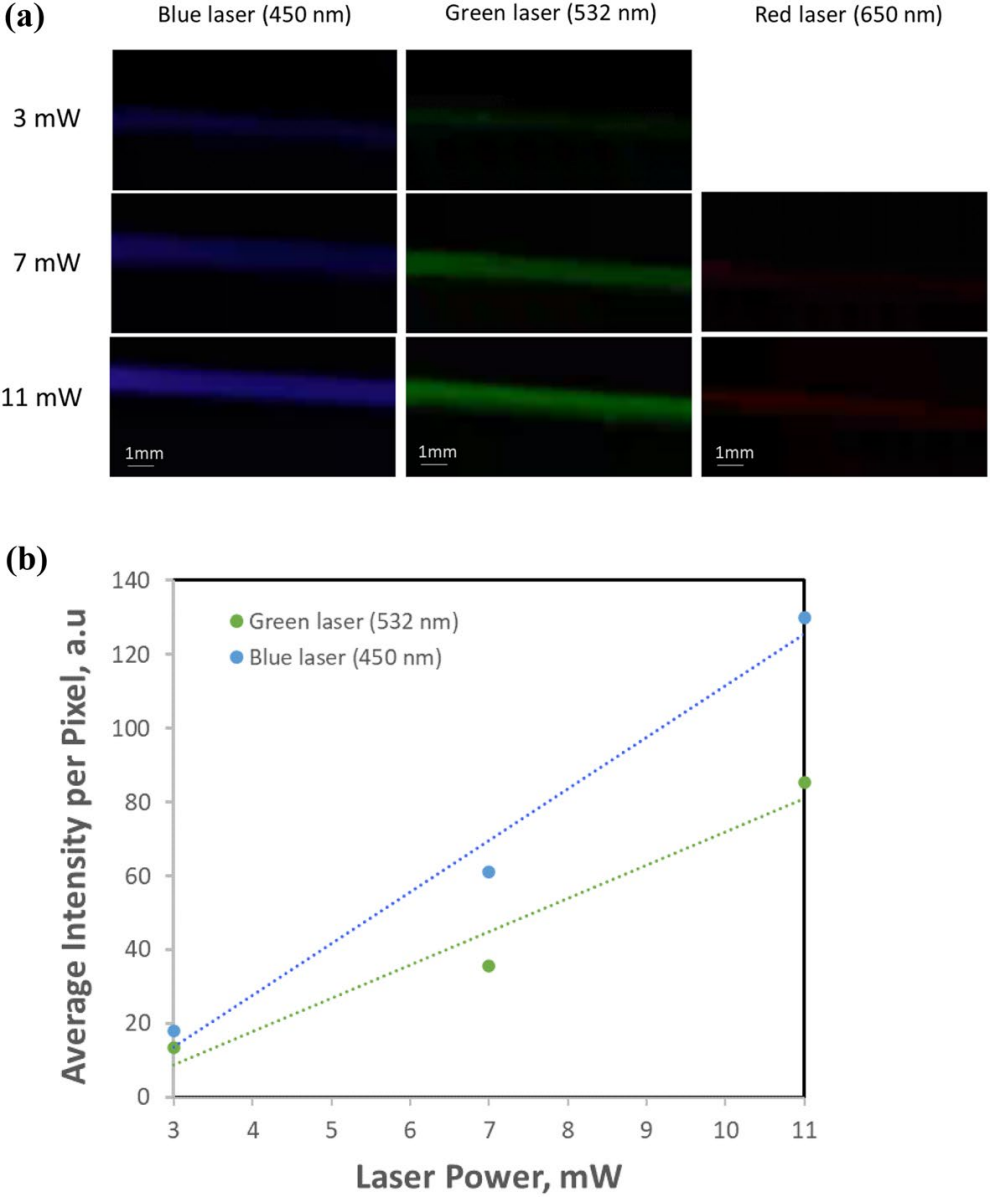
Light scattering images of pure water and the effect of laser power. (**a**) Light scattering images using lasers of nominal laser wavelengths at different laser powers. (**b**) The average intensity per pixel extracted from light scattering images using blue (450 nm) and green (532 nm) lasers at different laser powers. The water is purified, filled, and sealed into a clean glass bottle in a semiconductor fabrication clean room, and remains sealed during the light scattering test to minimize contamination. The laser power is adjusted using two wire-gride linear polarizers while keeping the polarization of the incident laser beam perpendicular to the earth or parallel to the imaging plane of the sensor. The average intensity per pixel is extracted from the light scattering images shown in (a) with a correction of quantum efficiency for the different colors.

Several experiments are performed to evaluate the brightness of the light scattering image under different laser irradiation. Figure 2a shows images of light scattering by pure water when blue, green, and red lasers with nominal wavelengths of 450 nm, 532 nm, and 650 nm are used at different laser powers. At 3 mW, none of the lasers can produce light scattering images distinguishable clearly from the dark background. The brightness of light scattering images for all lasers increases with the increase of the laser power. At 11 mW, the blue and the green lasers produce a clearly visible image of scattered light through the pure medium, but the red laser only produces a barely visible image. It is interesting to note that the brightness of the image resulting from the blue laser is about the same as that from the green laser, rather than 1.95 times brighter as calculated based on the inversely proportional to the 4^th^ power of the wavelength, ${1/\lambda }^{4}$, described in Eq. (2). This is because the brightness of light scattering images depends on also the image sensor and the optical system used for the test. Typically, color image sensors use a Bayer color sensing array configuration, in which each blue or red pixel is arranged with two green pixels in the array. Thus, green light produces double the light signal for the brightness if the light intensity for each pixel is the same. In addition, the quantum efficiency of blue pixels is typically lower than that of green pixels. Therefore, even though the intensity of light scattered by the medium using a green laser is lower than that using a blue laser, the image of green light scattering can look the same or brighter than the blue light scattering image.

Figure 2b shows the average intensity of light scattered by pure water at each sensing pixel when using blue and green lasers. The intensity at each pixel is extracted from the light scattering images and corrected using a quantum efficient. Referring to the data of similar image sensors, the quantum efficiency for the blue light is selected as 70% and the quantum efficiency of the green light is selected as 93% in the plot. The plot shows clearly that the light scattering intensity from the water is proportional to the power of the incident laser. It shows also that even though the light scattering image using the green laser is brighter than that using the blue laser, the average light scattering intensity per pixel using the blue laser is higher than that using the green laser. These experimental observations suggest that the brightness of the light scattering image perceived by an image sensor obeys the mechanism of Rayleigh scattering.

**Figure 3:**
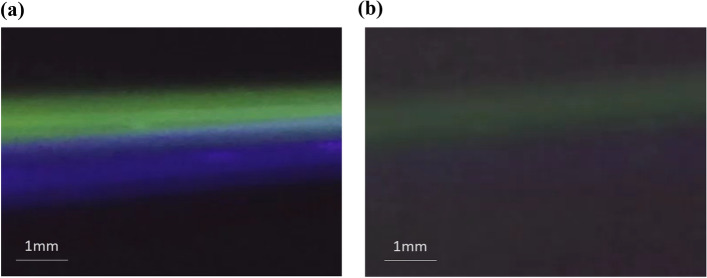
Light scattering images of pure water and air. A blue laser and a red laser are used simultaneously**.** (**a**) the light scattered by pure water, and (**b**) the light scattered by air.

Experiments are also performed to compare the light scattering by pure water and by particle-free air. In the test, each medium is irradiated with both blue and green laser beams at the same time. The power of these two lasers is adjusted to about 20 mW, and the laser beam diameter is adjusted to about 1 mm. A longer exposure time of the image sensor is used to make the image of the light scattered from the air more visible. As shown in Fig. 3, the light scattering by particle-free room air can be observed, but it is weaker than the light scattering by water. It is expected that, due to the lower index of refraction and concentration, higher laser power is required to enhance the light scattering image of air. According to the experimental observation, it is estimated that a green laser with a power greater than 100 mW may be needed to make the light scattering by air have a brightness similar to that of the water as shown in Fig. 3a. Although the laser power is quite high, it is still within the range of class 3B laser (5-500mW). Using light scattered by the air to detect nanoparticles suspended in the air still seems feasible.

Another important feature of Rayleigh scattering is that the scattering intensity varies with the angle $\varphi$ between the direction of oscillating dipole moment and the direction of the scattered light to be observed. When using a polarized light source, the intensity of a Rayleigh scattering will change with the change of ${sin}^{2}\varphi$ as shown in Eq. (2). In the era when only bulky lasers were available, it was common to fix the laser source in a position while rotating a photodetector, or using several photodetectors, around the laser for a light scattering measurement. For the application of detecting nanoparticles in a large container, it becomes impractical to rotate an image sensor around a laser beam. As the laser module becomes more compact, it is more convenient to rotate the direction of oscillating dipole moment through rotating the polarization of the incident laser beam relative to a fixed image sensor. It is more convenient to express the change of the angle $\varphi$ with the change of the angle ${\varphi }_{l.s}$ between the plane of linear polarization of incident laser and the plane of image sensing of the sensor. The angle $\varphi$ and the angle ${\varphi }_{l.s}$ are complementary, $\varphi +$ ${\varphi }_{l.s}=90^\circ$.

**Figure 4:**
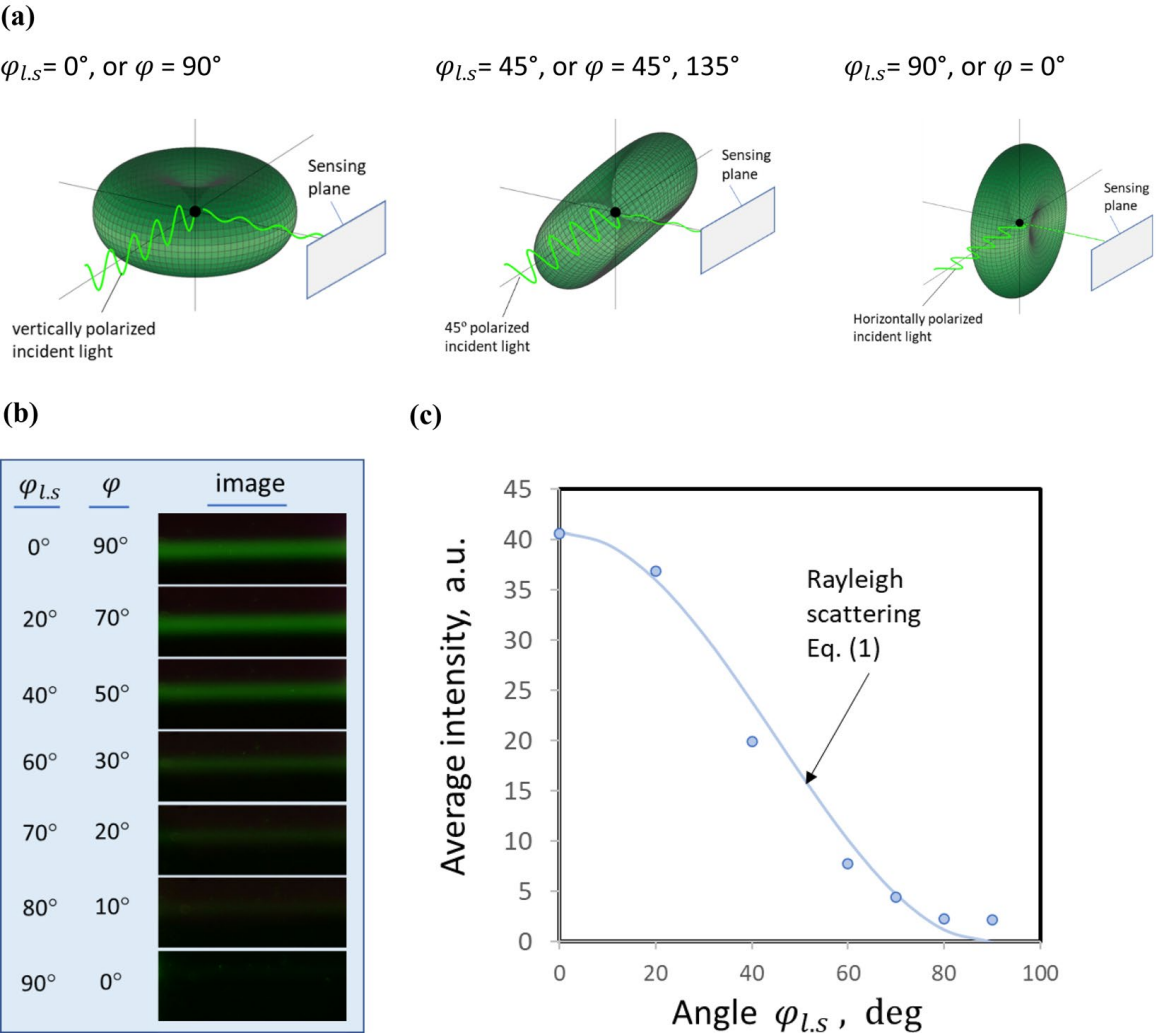
Effect of the angle ${\mathrm{\varphi }}_{\mathrm{l}.\mathrm{s}}$ between the incident light polarization plane and the sensing plane$.$ (**a**) The change of the induced light scattering profile with the incident light wave oscillation plane, or the incident light polarization plane, at several positions marked with corresponding ${\mathrm{\varphi }}_{\mathrm{l}.\mathrm{s}}$ and $\mathrm{\varphi }$ . (**b**) Change of brightness of light scattering image with the angles ${\mathrm{\varphi }}_{\mathrm{l}.\mathrm{s}}$ or $\mathrm{\varphi }$. (**c**) The plot of average intensity per sensing pixel extracted from the light scattering images shown in (b) as a function of the angle ${\mathrm{\varphi }}_{\mathrm{l}.\mathrm{s}}$, as well as a curve calculated based on Eq. (1).

Figure 4a shows 3-D curves of light scattering intensity profile based on the Eq. (1) when the plane in which incident light wave oscillates is parallel, 45°, and perpendicular to the plane that light scattering is sensed. Figure 4b shows images of light scattering by pure water when the angle $\varphi$ or ${\varphi }_{l.s}$ changes. The image sensor faces the laser beam at a fixed scattering angle $\theta$ of 90° relative to the laser propagation direction. The light scattering image of the beam is the brightest when the two planes are parallel ${\varphi }_{l.s}=0^\circ$, and the images become dimer and dimer as the two planes move from the parallel position toward a vertical position ${\varphi }_{l.s}=90^\circ$. Figure 4c plots the average intensities of the scattered light that are measured from light scattering images along the laser beams shown in Fig. 4b. A curve using $A\cdot {sin}^{2}\varphi$ or ${A\cdot sin}^{2}(90^\circ -{\varphi }_{l.s})$ based on Eqs. (1) or (2), is also plotted in Fig. 4c, where A is selected to match the average intensity value at ${\varphi }_{l.s}=0$. The plots show that the brightness of light scattering image changes with the change of incident light polarization plane, and the trend of the change is consistent with the Rayleigh scattering described in Eq. (1). Several other liquids and air are also tested at different angles of ${\varphi }_{l.s}$. Although the brightness of light scattering images for different media is different, the trend is the same.

### Light scattering images of nanoparticles suspended in a light scattering medium

**Figure 5:**
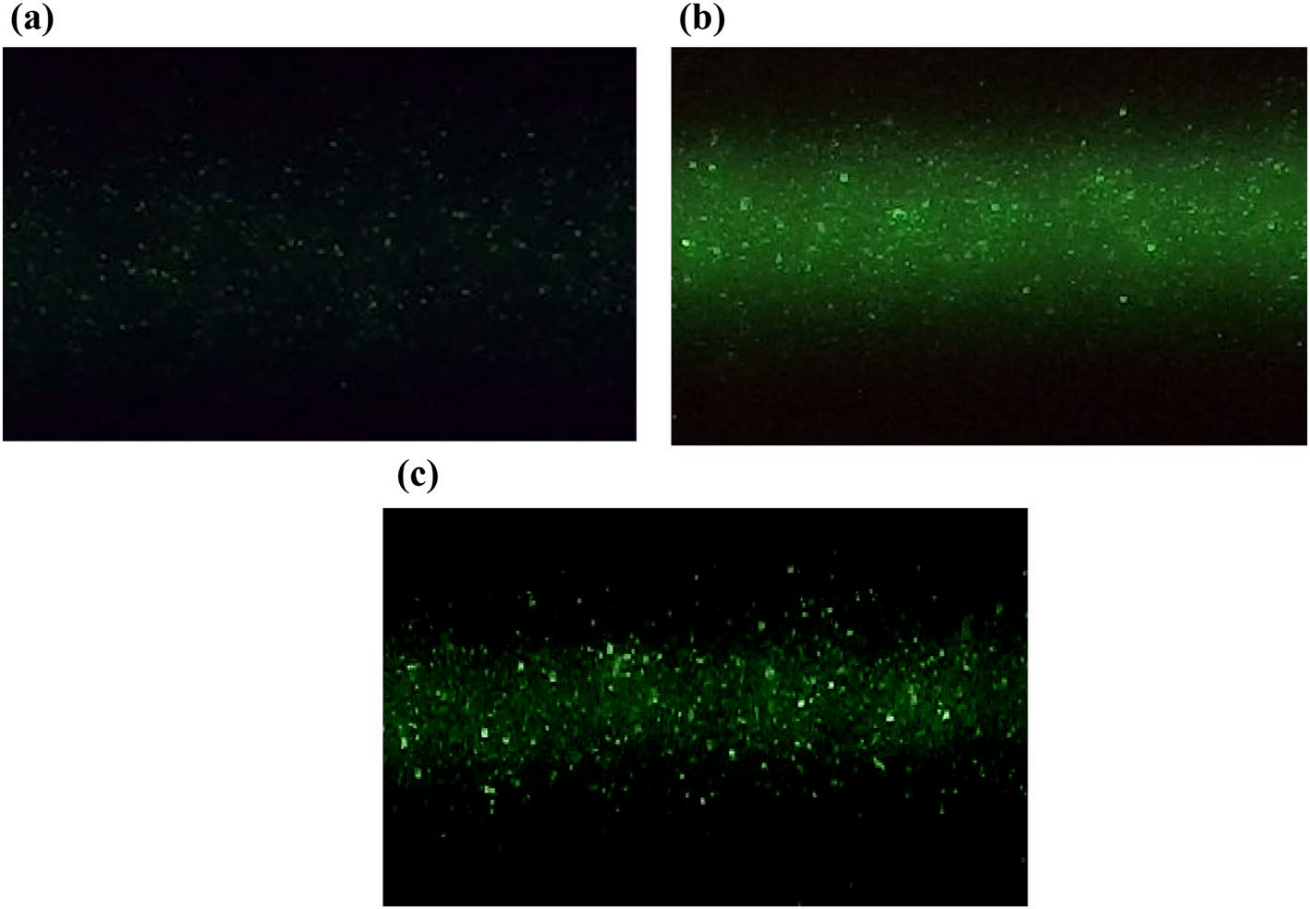
Images of light scattering by 40 nm SiO_2_ particles suspended in water. (**a**) Under the condition that water light scattering is barely visible. Incident laser power is 3 mW and the image of pure water is shown in Fig. 2(a). (**b**) Under the condition that the image of light scattering is clearly visible. Incident laser power is 15 mW. (**c**) Under the same condition as the (**b**), but the water light scattering is filtered out through an image sensor setting.

Figure 5a and b compare light scattering images of monodisperse 40 nm SiO_2_ nanosphere particles under conditions that the light scattering by water is almost invisible and clearly visible. It clearly shows that when the water light scattering is more visible, the particles look brighter and larger. In fact, the light scattered by water presents as a fine shiny background, and light scattered by the particles presents as moving bright dots on the shiny background. By setting the threshold intensity in the image sensor, the water scattering image can be filtered out without changing the nanoparticle light scattering image, as shown in Fig. 5c. When the light scattering by water is hidden from the image, all other light scattering effects remain the same. Therefore, the image still carries the enhancement effect from the light scattering medium on the scattering nanoparticles. The interference between the light scattered by the water and the light scattered by the particles may play a role in making the nanoparticles appear larger and brighter, as predicated by the third term in Eq. (3). However, more sophisticated experimental methods are needed to further confirm the interference effect.

**Figure 6:**
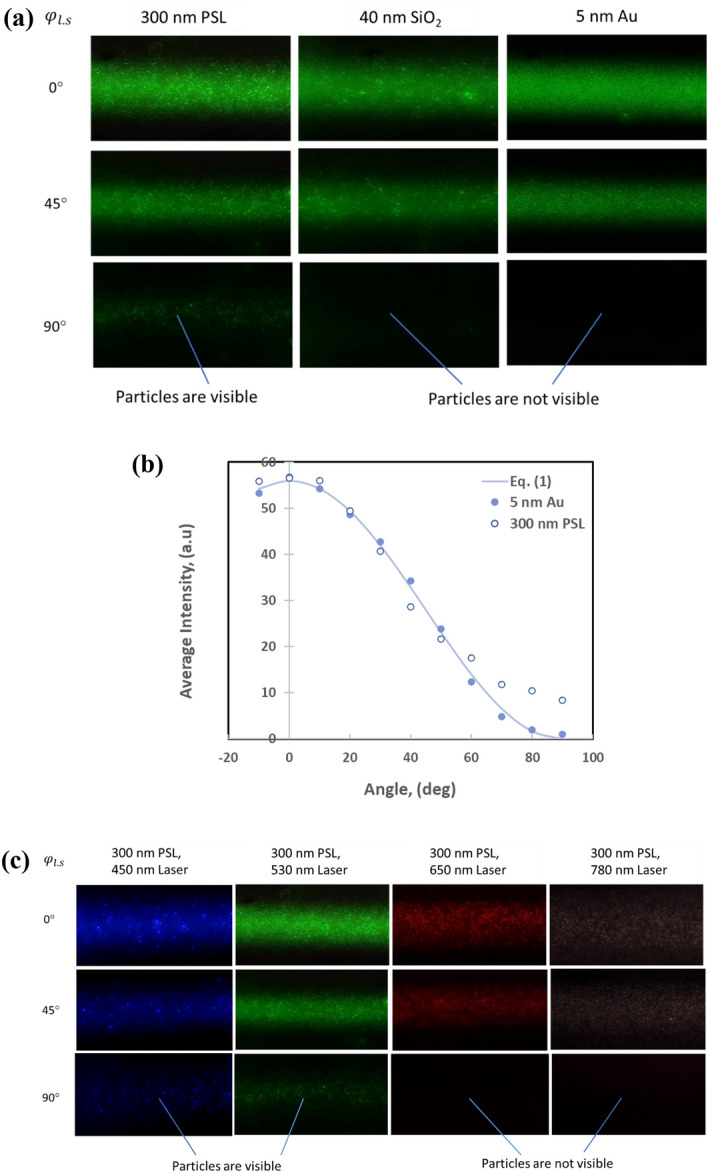
Effect of the incident light linear polarization on images of particle light scattering. (**a**) Images of the particles of different sizes when the angle ${\mathrm{\varphi }}_{\mathrm{l}.\mathrm{s}}$ is $0^\circ$, $45^\circ$, and $90^\circ$. Note that when ${\mathrm{\varphi }}_{\mathrm{l}.\mathrm{s}}=90^\circ$, the suspended 300 nm PSL particles are visible, and the 40 nm SiO_2_ and 5 nm Au particles are not visible. (**b**) A plot of the average intensity extracted from particle light scattering image for 5 nm Au particles and 300 nm PSL particles when the angle ${\mathrm{\varphi }}_{\mathrm{l}.\mathrm{s}}$ changes from -$10^\circ$ to $90^\circ$. (**c**) Images of light scattering of 300 nm PSL particles when using lasers of different wavelengths. The polarization of the incident light is rotated at three different angles relative to the sensing plane of the sensor. Note that when the angle is perpendicular ${\mathrm{\varphi }}_{\mathrm{l}.\mathrm{s}}=90^\circ$, the suspended 300 nm PSL particles are still visible when using blue and green lasers, but not visible when using red and infrared lasers.

If the particles are much smaller than the wavelength of the light source used for the particle detection, the intensity of scattered light should follow Rayleigh scattering as described in Eq. (1). The brightness of light scattered by particles should change when the polarization plane of the incident laser beam rotates against the sensing plane of the sensor. Standard particles suspended in several liquids are used for evaluation of the behavior. Figure 6a shows the images of light scattering by particles of 300 nm polystyrene latex (PSL) spheres, 40 nm SiO_2_ nanospheres, and 5 nm gold nanospheres at different angles of ${\varphi }_{l.s}$ suspended in water. In the test, the light scattered by water is visible, and the scattering angle $\theta$ relative to the laser propagation is fixed at 90°. If the light scattering is a Rayleigh scattering, the brightness of the particle scattering image should change as a function of ${\mathrm{\varphi }}_{\mathrm{l}.\mathrm{s}}$ or $\mathrm{\varphi }$. That is, when ${\varphi }_{l.s}$ is set at 90°, or the polarization plane and the sensing plane are perpendicular to each other, the scattered light intensity perceived by the sensor should be as the one with a dark background. It is observed in the experiment that, when ${\varphi }_{l.s}$ is 90°, suspended 300 nm PSL particles become dimmer but still visible, while 40 nm SiO_2_ and 5 nm Au particles are not visible. The 300 nm PSL particles behave differently from the smaller nanoparticles.

A more detailed measurement of particle light scattering with the change of angle ${\varphi }_{l.s}$ is performed using 300 nm PSL particles and 5 nm Au particles, as shown in Fig. 6(b). The light intensity is adjusted to make the intensity from both particles are about the same when ${\varphi }_{l.s}=0^\circ$, and the curve of $A\cdot {sin}^{2}\varphi$ or ${A\cdot sin}^{2}(90^\circ -{\varphi }_{l.s})$ is also plotted, where $A$ is select to make the value match the average intensity at ${\varphi }_{l.s}=0^\circ$ in the plot. The result shows that the light scattering by the 5 nm Au follows well with the Rayleigh scattering for all angles, while the light scattered by the 300 nm PSL particles starts to deviate from the Rayleigh scattering at the angle ${\varphi }_{l.s}$ above $70^\circ$. This can be explained that when the particle size is close to the wavelength of the incident laser, lights scattered by the particles no longer act as point sources and start to deviate from Rayleigh scattering into Mie scattering. This deviation is manifested in the direction where the emission due to the oscillating dipole moment is weak.

Light scattering by 300 nm PSL particles suspended in water is further tested with lasers of different wavelengths. When the angle ${\varphi }_{l.s}$ is 0°, the brightness of light scattering images from these lasers is adjusted to be similar. As shown in Fig. 6c, when ${\varphi }_{l.s}$ is 90°, the 300 nm PSL particles can be seen using blue (450 nm) and green (532 nm) lasers, but the particles cannot be seen using red (650 nm) and infrared (780 nm) lasers. Since the particle size is fixed, the longer the wavelength makes the particles relatively smaller, so the light scattering by the particles is more toward Rayleigh scattering. It further supports the explanation that when ${\varphi }_{l.s}$ is 90°, the visibility of the particles is dependent on whether the light scattering of the particles is the Rayleigh scattering or not. Since the visibility of particles is determined by how well the scattering from the particle follows the Rayleigh scattering, the feature can be utilized to distinguish nanometer particles from larger micrometer particles. Typically, the size of viruses is around 100 nm, while the size of bacteria is around 1 µm^13^. The method can roughly distinguish certain viruses and bacteria in a simple, non-intrusive way.

### Detection of suspended nanoparticles in a large container

**Figure 7:**
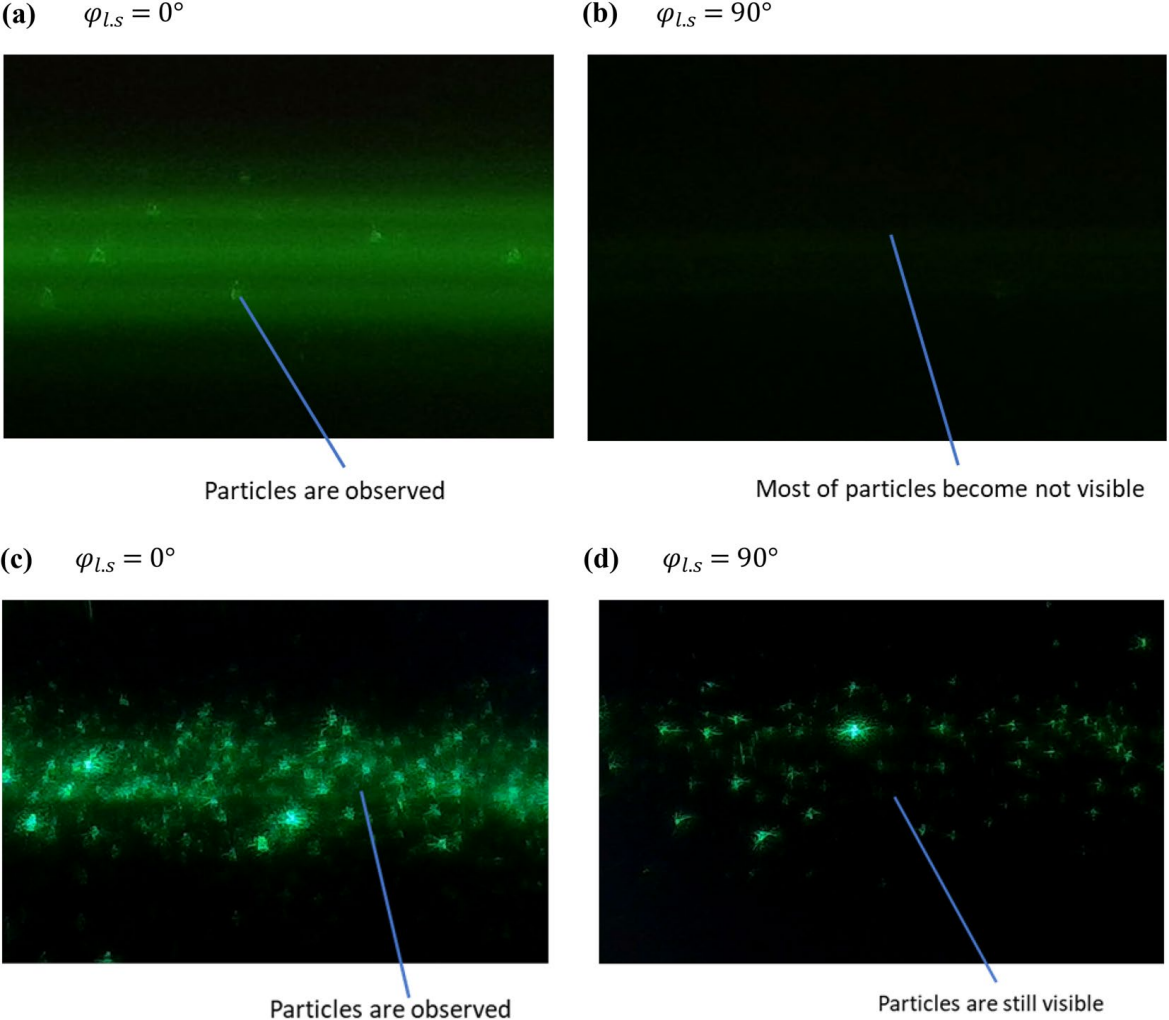
Detection of cleanliness of bottled drinking water using green (532 nm) laser. (**a**) and (**b**) a bottle of drinking water that remains sealed by its manufacturer during the test. (**a**) Using the laser with the polarization angle ${\mathrm{\varphi }}_{\mathrm{l}.\mathrm{s}}=0^\circ$. Suspended particles are observed as large moving dots on top of the light scattering image of water. (**b**) Using the laser with the polarization angle ${\mathrm{\varphi }}_{\mathrm{l}.\mathrm{s}}=90^\circ$. Most of the suspended particles become not visible. Therefore, the particles in the bottle are mainly the Rayleigh scattering particles, and they must be much smaller than the laser wavelength. (**c**) and (**d**) the bottled drinking water that is tested with a lower sensitivity setting one week after opening and drinking. (**c**) with the polarization angle ${\mathrm{\varphi }}_{\mathrm{l}.\mathrm{s}}=0^\circ$. More particles are observed. (**d**) with the polarization angle ${\mathrm{\varphi }}_{\mathrm{l}.\mathrm{s}}=90^\circ$. Many particles are still visible. Therefore, the particles in the open bottle are not Rayleigh scattering particles and must be much larger than the wavelength of the laser.

Encouraged by experimental observations mentioned above, a method is proposed to detect nanoparticles under the condition that the light scattering of the suspension medium is sufficiently intensified. The method is mainly for detecting nanoparticles suspended in large containers where optical microscope settings are not suitable. One of the applications tested is to check the cleanliness of bottled drinking water even without opening the cap sealed by the manufacturer. Figure 7 shows a test result using a bottle of purified water which is approximately 15 months away from its expiration date marked on the bottle. When the angle ${\varphi }_{l.s}$ between the polarization plane of the incident laser and the sensing plane of the image sensor is $0^\circ$, many particles are observed with water light scattering as a background, as shown in Fig. 7a. Particles can be easily recognized from the light scattering background from water since the particles are moving dots. When the angle ${\varphi }_{l.s}$ is rotated to close to 90°, although the laser power keeps as the same, most of the particles become invisible, as shown in Fig. 7b. Since most of the particles follow well the Rayleigh scattering equation as described in the previous section, the particles in the bottle are likely nanoparticles that are much smaller than the wavelength of the laser used for the detection. According to the ratio of the intensities of light scattered by particles and by water, the particles could be between 50 to 100 nm as a rough estimation. Since there are about 10 particles in the volume covered by the 5 mm long laser beam of around 1 mm diameter as shown in Fig. 7a, it is roughly estimated that the particle number concentration is less than 10^4^ particles per milliliter. The particle detection for the bottled water was repeated several times over several months. No significant change is observed between the detections. Therefore, the particles may be not from deterioration of the water cleanliness or the degradation of the bottle material. The particles are more likely to come from the manufacturing process. Several other bottles of drinking water from different manufactures and different storage conditions were tested. It shows a big difference in terms of particle concentration and particle size. Some of the particles are likely coming from bottle materials, such as micro-plastics, and some of the particles are likely living organisms.

One of the bottles of drinking water is checked again one week after drinking half of the water in the bottle. The initial condition of the bottled drinking water is like the one shown in Fig. 7a. After drinking, the bottle was not tightly capped and was placed in a warm room. When performing the particle detection at the angle ${\varphi }_{l.s}=0^\circ$, much more particles are observed, as shown in Fig. 7c. When performing the particle detection at the angle ${\varphi }_{l.s}=90^\circ$, there are still a lot of particles are visible, as shown in Fig. 7d. Therefore, many of the particles in the bottle observed at the time are much larger than the wavelength of the incident laser. These particles are likely microorganisms that are multiplied and grown after the cap is opened and may be harmful to the human body.

Detection of particles packed originally or collected in different transparent containers in different liquids or air is also performed with interesting observations. Since the method does not require the transfer of the media from one container to another container and does not require the particles located in a small optical focal space, it can be mounted or equipped with more flexibility. It will lead to many unique applications in the future.

## Discussion

The intensified light scattering of the suspension medium can be intentionally utilized for the detection of nanoparticles. When light scattering by the medium is intensified, the light scattering by the particles suspended in the medium becomes more intensified, and constructive interference may occur between the light scattered by the particles and the light scattered by the medium. When using a pulse detection approach, the benefits of the light scattering medium may be limited, because strong light scattering by the medium may result in a lower signal-to-noise ratio or a loss in the dynamic range of the detection. However, when using the image detection approach, this is no longer a problem, because the image detection approach is more like an interferometer, where the reference and signal are on the same image. On the other hand, the intensified light scattering of the medium can also be the result of using lasers of a higher power. As indicated in Eq. (1), a higher incident laser power or intensity is needed to obtain a stronger light scattering from a particle for detecting smaller and smaller particles. The high incident laser power results in a stronger light scattering by the medium. As the work shows, the medium light scattering should not be something to worry about or avoid, especially when using the image detection method. Lasers used in the work are in the low power range of Class 3B lasers. Within the same laser safety category, there is still a lot of room for the use of higher power lasers to allow suspended nanoparticles to be seen more clearly.

The particle detection approach based on light scattering image detection can be further extended to particle analysis through development work. Particle concentration can be measured through brightness measurement as the concentration correlates with the total light scattering intensity shown in Eqs. (5) and (6). Under certain conditions, the concentration of suspended particles can be directly determined through digital image analysis of a picture or movie of particles. For example, several algorithms using open-cv with Python^14^, available for computer vision or machine learning, can be utilized to determine particle count or particle number concentration from light scattering images of particles. In addition, it is possible to further determine the particle size distribution. Several adjustable measurement functions can be utilized to determine the relationship between particle concentration and particle size distribution as shown in Eq. (6). The adjustable measurement functions include tuning the scattering angle relative to the dipole moment direction, displaying selectively images of light intensities through image sensor settings, and screening light scattering patterns through an image analysis algorithm. The size of suspended particles can be also determined from the difference in their thermal velocities. As the light scattered by the medium becomes strong, the difference in lights scattered by particles of different sizes also increases. Integrating the unique measurement functions provided by the image detection with the light scattering of the suspension medium can further develop a new method for analyzing the size distribution of suspended nanoparticles.

Overall, an image detection method is proposed to detect suspended nanoparticles that are much smaller than the wavelengths of visible light. The method utilizes the intensified light scattering of the suspension medium to make the nanoparticles easier to detect by an image sensor. Since this method does not rely on optical lenses to focus or observe light in a small space, it is more flexible for the detection of particles suspended in different conditions. As demonstrated experimentally, the method can be used to directly detect suspended nanoparticles contained in large containers, such as sealed glass or plastic bottles, and distinguish between small nanoparticles and large microparticles. It provides a new direction for the further development of new particle detection technologies as well as many new applications.

## Methods

### Experimental setup

Experiments performed in the work use compact 12 mm diameter laser modules of several selected wavelengths. The laser power ranges from 5 to 50 mW. The laser modules are obtained from online stores such as Thurlab, Amazon, and eBay with specified wavelengths and powers. The laser beam passes through a linear polarizer and iris of adjustable diameter from 0 to 5 mm. The output power of the laser to the water was adjusted using two polarizers align or against each other. The laser power incident to the sample is checked using a portable laser power meter (Sanawa, Mobiken LP1). The laser and polarizer are mounted on a rotatable station so that it can change the angle between the beam polarization plane and imaging plane while keeping the incident power the same. No condenser lens is used. In the system, the sensor array used is a 2 MP Sony IMX 291 image sensor with a pixel size of 2.9 $\mu m$ × 2.9 $\mu m$. The lens at the front of the image sensor is a telephoto lens of variable focal length, instead of an objective lens of a high numerical aperture. The sensor is located at an angle of 90° relative to the incident beam, and the angle can be adjusted to another angle if needed. The distance between the entrance pupil of the telephoto lens and the laser beam is adjustable, typically between 15 to 50 mm. The suspended nanoparticles can be contained in a cuvette, beaker, or bottle of a diameter up to 100 mm.

The water used for testing medium light scattering and particle sample preparation comes from a semiconductor fabrication cleanroom purified for wafer processes. The clean air used is the air passing through a H13 HEPA filter. The standard particles used in the experiment are 5 nm gold nanospheres (nanoComposix, AUCN5-100 M), 40 nm silicon oxide nanospheres (Microspheres-Nanospheres, 140,108–10), and 300 nm PSL spheres (Thermo Scientific, 3300A). The particles were diluted in water through a transfer using droplets from the original containers or using a cleaned syringe without any syringe filter to avoid any undesired contamination. When the incident laser is set at the angle ${\varphi }_{l.s}=0^\circ$, the particle number concentration in the samples is adjusted to obtain similar light scattering intensities.

Light scattering from particles and medium. Without the presence of the large nanoparticle, the total intensity of scattered light by n small particles is

$$\begin{aligned} I_{nM} & = \frac{1}{2}\varepsilon_{0} c\left| {\mathop \sum \limits_{i = 1}^{n} E_{s,m,i} } \right|^{2} = \frac{1}{2}\varepsilon_{0} cE_{0}^{2} \mathop \sum \limits_{i = 1}^{n} A_{m,i} e^{{{\text{i}}\left( {\omega t - \varphi_{S,i} } \right)}} \mathop \sum \limits_{j = 1}^{n} A_{m,j} e^{{ - {\text{i}}\left( {\omega t - \varphi_{m,j} } \right)}} \\ & = \frac{1}{2}\varepsilon_{0} cE_{0}^{2} \mathop \sum \limits_{i = 1}^{n} \mathop \sum \limits_{j = 1}^{n} A_{m,i} A_{m,j} e^{{ - {\text{i}}\left( {\varphi_{m,j} - \varphi_{m,i} } \right)}} \\ & = \frac{1}{2}\varepsilon_{0} cE_{0}^{2} \mathop \sum \limits_{i = 1}^{n} \mathop \sum \limits_{j = 1}^{n} A_{m,i} A_{m,j} \left( {\cos \left( {\varphi_{m,j} - \varphi_{m,i} } \right) - i\sin \left( {\varphi_{m,j} - \varphi_{m,i} } \right)} \right) \\ \end{aligned}$$

Since the $\sin \left( {\varphi_{m,j} - \varphi_{m,i} } \right) = - \sin \left( {\varphi_{m,j} - \varphi_{m,i} } \right)$, when $i \ne j$, $\sin \left( {\varphi_{m,j} - \varphi_{m,i} } \right) = 0$, when $i = j$, it leads to Eq. (7) as

7 $${I}_{nM}=\frac{1}{2}{\varepsilon }_{0}c{{E}_{0}}^{2}\sum_{i=1}^{n}\sum_{j=1}^{n}{A}_{m,i}{A}_{m,j}\mathrm{cos}\left({\varphi }_{m,i}-{\varphi }_{m,j}\right)$$

When both the large particle and *n* small light scatters are present, the total intensity to reach the detector $I_{ 1L,nS}$ is

8 $$\begin{aligned} I_{ p,nM} & = \frac{1}{2}\varepsilon_{0} cE_{0}^{2} \left( {A_{L} e^{{{\text{i}}\left( {\omega t - \varphi_{p} } \right)}} + \mathop \sum \limits_{i = 1}^{n} A_{S,i} e^{{{\text{i}}\left( {\omega t - \varphi_{m,i} } \right)}} } \right)\left( {A_{L} e^{{ - {\text{i}}\left( {\omega t - \varphi_{p} } \right)}} + \mathop \sum \limits_{i = 1}^{n} A_{S,i} e^{{ - {\text{i}}\left( {\omega t - \varphi_{m,i} } \right)}} } \right) \\ & = \frac{1}{2}\varepsilon_{0} cE_{0}^{2} \left( {A_{p}^{2} + \mathop \sum \limits_{i = 1}^{n} A_{m,i} e^{{{\text{i}}\left( {\omega t - \varphi_{m,i} } \right)}} \mathop \sum \limits_{j = 1}^{n} A_{S,j} e^{{ - {\text{i}}\left( {\omega t - \varphi_{m,j} } \right)}} + A_{L} e^{{{\text{i}}\left( {\omega t - \varphi_{p} } \right)}} \mathop \sum \limits_{i = 1}^{n} A_{S,i} e^{{ - {\text{i}}\left( {\omega t - \varphi_{m,i} } \right)}} + A_{L} e^{{ - {\text{i}}\left( {\omega t - \varphi_{p} } \right)}} \mathop \sum \limits_{i = 1}^{n} A_{S,i} e^{{{\text{i}}\left( {\omega t - \varphi_{m,i} } \right)}} } \right) \\ & = I_{p} + \frac{1}{2}\varepsilon_{0} cE_{0}^{2} \mathop \sum \limits_{i = 1}^{n} \mathop \sum \limits_{j = 1}^{n} A_{m,i} A_{m,j} e^{{{\text{i}}\left( {\varphi_{m,i} - \varphi_{m,j} } \right)}} + \frac{1}{2}\varepsilon_{0} cE_{0}^{2} \mathop \sum \limits_{i = 1}^{n} A_{p} A_{m,i} e^{{{\text{i}}\left( {\varphi_{m,i} - \varphi_{p} } \right)}} + \frac{1}{2}\varepsilon_{0} cE_{0}^{2} \mathop \sum \limits_{i = 1}^{n} A_{p} A_{m,i} e^{{ - {\text{i}}\left( {\varphi_{m,i} - \varphi_{p} } \right)}} \\ & = I_{p} + I_{nM} + \frac{1}{2}\varepsilon_{0} cE_{0}^{2} \left[ {\mathop \sum \limits_{i = 1}^{n} A_{p} A_{m,i} \cos \left( {\varphi_{m,i} - \varphi_{p} } \right) + \mathop \sum \limits_{i = 1}^{n} A_{p} A_{m,i} \cos \left[ { - \left( {\varphi_{m,i} - \varphi_{p} } \right)} \right] + i\mathop \sum \limits_{i = 1}^{n} A_{p} A_{m,i} \sin \left( {\varphi_{m,i} - \varphi_{p} } \right) - i\mathop \sum \limits_{i = 1}^{n} A_{L} A_{S,i} \sin \left( {\varphi_{m,i} - \varphi_{p} } \right)} \right] \\ & = I_{p} + I_{nM} + 2I_{p} \mathop \sum \limits_{i = 1}^{n} \frac{{A_{m,i} }}{{A_{p} }}\cos \left( {\varphi_{m,i} - \varphi_{p} } \right) \\ \end{aligned}$$
